# Association Between the C-Reactive Protein-Albumin-Lymphocyte (CALLY) Index and Tissue-Confirmed Cytomegalovirus Colitis in Patients with Active Ulcerative Colitis

**DOI:** 10.3390/diagnostics16142202

**Published:** 2026-07-14

**Authors:** Mehmet Kursad Keskin, Tufan Teker, İmran Sağlık, Nesrin Uğraş, Fatih Eren, Mahmut Enver Dolar

**Affiliations:** 1 Department of Gastroenterology, Faculty of Medicine, Bursa Uludağ University, Bursa 16059, Türkiye; tufanteker@hotmail.com (T.T.); edolar@uludag.edu.tr (M.E.D.); 2 Department of Medical Microbiology, Faculty of Medicine, Bursa Uludağ University, Bursa 16059, Türkiye; imransaglik@uludag.edu.tr; 3 Department of Medical Pathology, Faculty of Medicine, Bursa Uludağ University, Bursa 16059, Türkiye; nesrinugras@uludag.edu.tr

**Keywords:** ulcerative colitis, cytomegalovirus, tissue-confirmed CMV colitis, CALLY index, inflammation-based biomarker, risk stratification

## Abstract

**Background**: Cytomegalovirus (CMV) reactivation is a clinically important complication in patients with active ulcerative colitis (UC) and has been associated with severe disease activity, treatment refractoriness, and adverse outcomes. The C-reactive protein–albumin–lymphocyte (CALLY) index is a composite biomarker reflecting systemic inflammation, nutritional status, and immune competence. However, its potential role in identifying patients with tissue-confirmed CMV colitis in UC has not been investigated. **Methods:** This retrospective, single-center cross-sectional study included 82 adults with active ulcerative colitis who underwent tissue evaluation for CMV infection between January 2018 and October 2023. CMV positivity was defined by histopathological and/or immunohistochemical confirmation of CMV infection in colonic biopsy specimens. The CALLY index was calculated as serum albumin × absolute lymphocyte count/C-reactive protein. Receiver operating characteristic (ROC) analysis was performed to evaluate diagnostic performance, and logistic regression analyses were used to identify independent predictors of tissue-confirmed CMV colitis. **Results:** Eighty-two patients were included, of whom 28 (34.1%) had tissue-confirmed CMV colitis. CMV-positive patients were older and had higher Mayo scores, more extensive disease, lower serum albumin levels, and higher fecal calprotectin concentrations than CMV-negative patients. Although median CALLY index values were numerically higher in CMV-positive patients, the difference did not reach statistical significance (*p* = 0.136). ROC analysis demonstrated modest discriminative performance for the CALLY index (AUC = 0.601, 95% CI: 0.472–0.722). The optimal CALLY cut-off value of 2838 was derived from the present dataset using the Youden index and yielded a sensitivity of 82.1% and a specificity of 50.0%. In multivariable logistic regression analysis, CALLY ≥ 2838 remained independently associated with tissue-confirmed CMV colitis (OR = 32.09, 95% CI: 3.27–315.39, *p* = 0.003). **Conclusions:** The CALLY index demonstrated limited standalone diagnostic performance for identifying CMV colitis in active UC. However, a CALLY value ≥ 2838 was independently associated with tissue-confirmed CMV positivity and may provide complementary information for risk stratification alongside established clinical and inflammatory markers.

## 1. Introduction

Ulcerative colitis (UC) is a chronic relapsing inflammatory bowel disease characterized by recurrent episodes of mucosal inflammation and progressive intestinal damage. Despite substantial advances in therapeutic strategies, many patients continue to experience severe disease flares, hospitalization, corticosteroid dependence, treatment failure, and colectomy. In addition to uncontrolled intestinal inflammation, infectious complications represent an important determinant of disease burden and clinical outcomes in UC. Patients with inflammatory bowel disease are particularly susceptible to opportunistic infections because of underlying immune dysregulation and the widespread use of corticosteroids, immunomodulators, and biologic therapies [1]. Among these infections, cytomegalovirus (CMV) has emerged as one of the most clinically significant pathogens, particularly in patients with severe or treatment-refractory disease [2,3].

Cytomegalovirus (CMV) reactivation represents one of the most clinically relevant challenges in patients with active UC. CMV is a ubiquitous β-herpesvirus that establishes lifelong latency after primary infection and may reactivate under conditions of immune dysregulation or intense inflammatory activity [2]. Several studies have identified severe disease activity, extensive colitis, corticosteroid exposure, and immunosuppressive therapy as major risk factors for CMV reactivation in UC [4,5]. Moreover, CMV infection has been associated with steroid refractoriness, colectomy, and adverse clinical outcomes, particularly among patients with acute severe ulcerative colitis [3,4].

Despite growing evidence supporting the clinical relevance of CMV infection in UC, its exact pathogenic role remains controversial. While some investigators consider CMV reactivation to be merely a bystander phenomenon reflecting severe underlying inflammation, others suggest that CMV may actively contribute to disease progression by amplifying mucosal immune responses, increasing tissue injury, impairing mucosal healing, and reducing responsiveness to medical therapy [6,7,8,9]. This ongoing debate has important clinical implications because differentiating patients with clinically significant CMV colitis from those with severe inflammatory activity alone remains challenging in routine practice. Consequently, reliable tools capable of identifying patients at increased risk of CMV colitis are needed to support timely diagnostic evaluation and therapeutic decision-making.

Current diagnostic approaches for CMV colitis rely primarily on endoscopic biopsy, histopathological examination, immunohistochemistry, and tissue CMV DNA quantification. Although these methods provide high diagnostic accuracy, they are invasive, costly, and not always feasible in all patients with active UC [2,4]. Furthermore, distinguishing clinically relevant CMV colitis from severe inflammatory activity alone remains challenging, and no universally accepted noninvasive biomarker is currently available. Previous studies have proposed CMV DNA quantification and clinical prediction models as potential tools for risk stratification; however, their routine clinical applicability remains limited [10,11]. Therefore, there is a continuing need for simple, inexpensive, and readily available biomarkers capable of identifying patients at increased risk of CMV colitis.

Recently, increasing attention has been directed toward composite inflammation-based biomarkers that integrate multiple biological pathways involved in disease progression. The C-reactive protein–albumin–lymphocyte (CALLY) index is a composite inflammation-based biomarker that combines systemic inflammation, nutritional status, and immune competence into a single parameter. Originally developed and validated as a prognostic indicator in oncology, the CALLY index has been associated with survival outcomes in gastric and colorectal cancers and has also shown potential utility in non-malignant inflammatory conditions [12,13,14]. Given that its individual components have been linked to disease severity and adverse outcomes in ulcerative colitis [15,16,17], the CALLY index may represent a practical and readily available tool for identifying patients at increased risk of CMV colitis.

The biological rationale for evaluating the CALLY index in CMV colitis is supported by the pathophysiology of CMV reactivation in ulcerative colitis. CMV reactivation typically occurs in patients with severe mucosal inflammation, impaired nutritional status, and dysregulated cellular immunity. Because the CALLY index integrates C-reactive protein as a marker of systemic inflammation, serum albumin as an indicator of nutritional and inflammatory status, and peripheral lymphocyte count as a surrogate of immune competence, it may reflect the biological milieu that predisposes patients with active ulcerative colitis to clinically significant CMV reactivation, thereby providing a biologically plausible rationale for evaluating the CALLY index in this clinical setting [5,6,18,19,20].

Inflammation-based indices derived from routinely available laboratory parameters have increasingly been investigated as practical tools for assessing disease severity, predicting clinical outcomes, and supporting risk stratification in ulcerative colitis [15,16,17,21]. Nevertheless, the potential role of the CALLY index in identifying patients at risk for CMV colitis has not been explored. To the best of our knowledge, no previous study has specifically evaluated the relationship between the CALLY index and tissue-confirmed CMV colitis in patients with active UC. Therefore, the aim of the present study was to investigate the association between the CALLY index and tissue-confirmed CMV colitis and to evaluate the diagnostic performance of the CALLY index for identifying patients with tissue-confirmed CMV colitis.

## 2. Materials and Methods

### 2.1. Study Design and Patient Population

This retrospective, cross-sectional study was conducted at the Department of Gastroenterology, Bursa Uludağ University Faculty of Medicine, Türkiye. The study protocol was approved by the Bursa Uludağ University Clinical Research Ethics Committee (Approval No. 2023-10/36; 10 October 2023), and the study was conducted in accordance with the principles of the Declaration of Helsinki.

The institutional inflammatory bowel disease database was retrospectively reviewed to identify all adult patients with an established diagnosis of ulcerative colitis who were followed between January 2018 and October 2023. During the study period, 500 patients with ulcerative colitis were identified.

Tissue-based cytomegalovirus (CMV) evaluation was not routinely performed in all patients with active ulcerative colitis. Instead, CMV investigation was requested according to the treating gastroenterologist’s clinical judgment in patients with active ulcerative colitis who fulfilled predefined clinical criteria suggestive of CMV colitis, in accordance with contemporary clinical practice and current international recommendations. These predefined clinical criteria included steroid-refractory disease, inadequate response to medical therapy, extensive colitis, and/or endoscopic findings suggestive of CMV infection.

**Figure 1:**
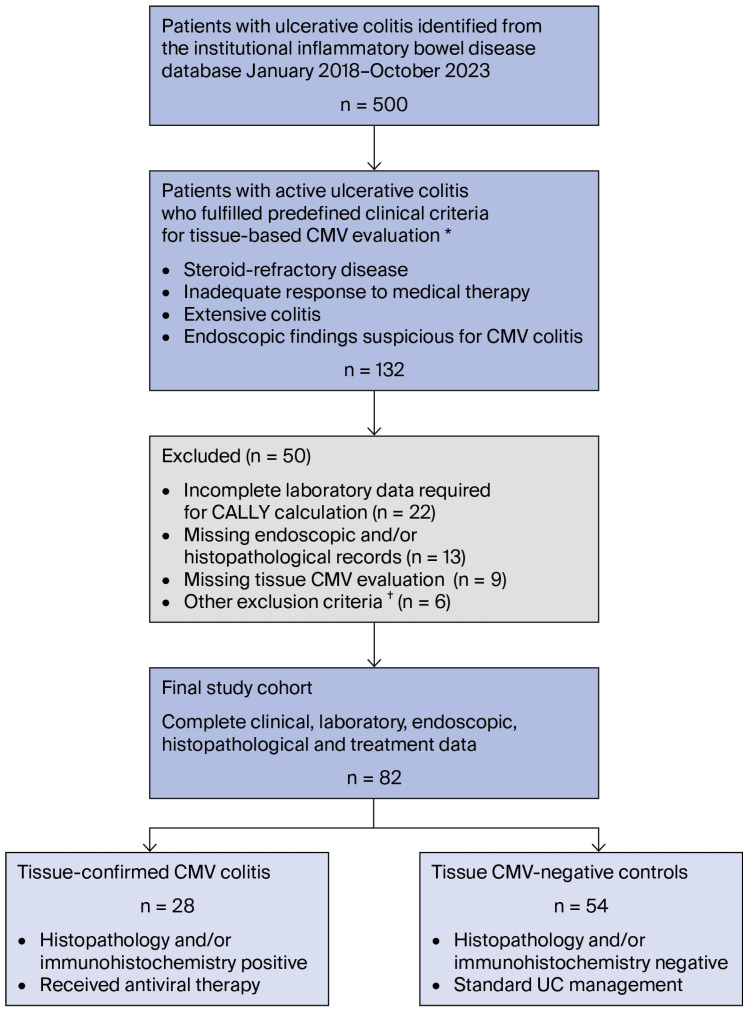
Flow diagram of patient selection and study cohort. * Tissue CMV evaluation was not routinely performed in all patients with active ulcerative colitis. Testing was requested according to the treating gastroenterologist’s clinical judgment in patients with steroid-refractory disease, inadequate response to medical therapy, extensive colitis, or endoscopic findings suggestive of CMV infection. ^†^ Other exclusion criteria included concomitant malignancy, HIV infection, previous solid-organ or hematopoietic stem-cell transplantation, and infectious colitis caused by pathogens other than CMV.

Consequently, 132 patients underwent tissue-based CMV evaluation. The patient selection process is summarized in Figure 1. Patients were eligible for inclusion if they were 18 years of age or older, had an established diagnosis of ulcerative colitis based on standard clinical, endoscopic, and histopathological criteria, underwent colonoscopic or flexible sigmoidoscopic evaluation during active disease, had available tissue-based CMV assessment, and had complete laboratory data required for calculation of the C-reactive protein–albumin–lymphocyte (CALLY) index.

Patients were excluded because of incomplete laboratory data required for CALLY index calculation (*n* = 22), missing endoscopic and/or histopathological records (*n* = 13), missing tissue CMV evaluation (*n* = 9), or other exclusion criteria (*n* = 6), including concomitant malignancy, human immunodeficiency virus infection, previous solid-organ or hematopoietic stem-cell transplantation, or infectious colitis caused by pathogens other than CMV.

After the application of the predefined eligibility criteria, 82 patients constituted the final study cohort, including 28 patients with tissue-confirmed CMV colitis and 54 CMV-negative controls (Figure 1). Accordingly, the CMV positivity rate observed in the present study reflects a clinically selected high-risk population undergoing tissue-based CMV evaluation rather than the overall prevalence of CMV colitis among patients with ulcerative colitis.

### 2.2. Assessment of Cytomegalovirus Infection

Tissue-based cytomegalovirus (CMV) evaluation was performed in all patients included in the final study cohort. These patients represented a clinically selected high-risk population with active ulcerative colitis who fulfilled predefined clinical criteria for tissue CMV investigation, consistent with current ECCO and AGA recommendations for the evaluation of suspected CMV colitis in patients with active ulcerative colitis. Tissue CMV evaluation was requested in patients with steroid-refractory disease, inadequate response to medical therapy, severe or extensive colitis, and/or endoscopic findings raising suspicion for CMV colitis.

During colonoscopy or flexible sigmoidoscopy, multiple targeted biopsy specimens were obtained from the ulcer base, ulcer edge, and the most severely inflamed mucosal areas considered suspicious for CMV infection by the endoscopist. In accordance with our institutional diagnostic protocol, all biopsy specimens routinely underwent histopathological examination, CMV immunohistochemical (IHC) staining, and tissue CMV polymerase chain reaction (PCR) analysis. Consequently, histopathology, IHC, and tissue CMV-PCR results were available for all 82 patients included in the study.

The reference standard for CMV colitis was tissue confirmation by histopathological identification of characteristic CMV cytopathic inclusions and/or positive CMV immunohistochemical staining. Tissue CMV-PCR findings were recorded as supportive virological evidence but were not used as the sole diagnostic criterion because positive tissue PCR may reflect latent viral infection or low-level viral replication without histological evidence of tissue-invasive disease. Accordingly, patients with isolated tissue CMV-PCR positivity in the absence of histopathological or immunohistochemical confirmation were classified as CMV-negative for the purposes of this study.

The CMV-negative control group consisted of patients with active ulcerative colitis who fulfilled the same predefined clinical criteria for tissue CMV investigation, underwent identical endoscopic biopsy sampling and tissue-based CMV assessment, but demonstrated no histopathological or immunohistochemical evidence of tissue-invasive CMV infection.

### 2.3. Calculation of the CALLY Index

The C-reactive protein–albumin–lymphocyte (CALLY) index was calculated using laboratory measurements obtained during the active disease episode before initiation of antiviral treatment.

The CALLY index was calculated according to the following formula:

CALLY index = (Serum albumin [g/L] × Absolute lymphocyte count [/µL])/C-reactive protein [mg/L].

Serum albumin concentration (g/L), absolute lymphocyte count (/µL), and serum C-reactive protein (mg/L) were obtained from blood samples collected at the time of hospitalization or endoscopic evaluation before antiviral therapy.

To facilitate reproducibility, a representative calculation is provided. For example, a patient with a serum albumin concentration of 30 g/L, an absolute lymphocyte count of 2000/µL, and a CRP level of 20 mg/L would have a CALLY index of 3000, calculated as (30 × 2000)/20.

No patients included in the study had a CRP value of zero or below the analytical detection limit; therefore, no correction factor or imputation procedure was required. In addition, CALLY values were analyzed as originally calculated without logarithmic transformation, truncation, or winsorization.

### 2.4. Clinical and Laboratory Data Collection

**The following variables were extracted from institutional records:**

**Demographic Variables**

* Age;
* Sex;
* Body mass index;
* Smoking status;
* Disease duration.

**Disease Characteristics**

* Total Mayo score;
* Mayo Endoscopic Subscore (MES);
* Montreal disease extent classification (E1, E2, and E3).

**Laboratory Parameters**

* C-reactive protein (CRP);
* Erythrocyte sedimentation rate (ESR);
* Serum albumin;
* Hemoglobin;
* Total leukocyte count;
* Absolute lymphocyte count;
* Platelet count;
* Fecal calprotectin;
* Tissue CMV-DNA level (copies/mg tissue).

**Treatment Variables**

* Corticosteroid exposure;
* Azathioprine treatment;
* Anti-tumor necrosis factor (anti-TNF) therapy;
* Vedolizumab therapy;
* Ustekinumab therapy.

Treatment exposure was defined as receipt of the respective medication at the time of the active ulcerative colitis episode leading to the index endoscopic evaluation. Treatment status was determined from institutional medical records and reflected ongoing therapy immediately before or at the time of tissue CMV assessment.

### 2.5. Study Outcomes

The primary outcome of the study was tissue-confirmed CMV colitis.

**Secondary outcomes included:**

* Comparison of CALLY index values between CMV-positive and CMV-negative patients;
* Determination of the optimal CALLY cut-off value for identifying CMV-positive patients;
* Evaluation of the diagnostic performance of the CALLY index using receiver operating characteristic (ROC) analysis;
* Identification of independent predictors of tissue-confirmed CMV colitis.

### 2.6. Statistical Analysis

Statistical analyses were performed using IBM SPSS Statistics version 26.0 (IBM Corp., Armonk, NY, USA). The distribution of continuous variables was assessed using the Kolmogorov–Smirnov test and visual inspection of histograms. Continuous variables are expressed as median and interquartile range (IQR), whereas categorical variables are presented as frequencies and percentages.

Comparisons between CMV-positive and CMV-negative patients were performed using the Mann–Whitney U test for continuous variables and the chi-square test or Fisher’s exact test for categorical variables, as appropriate.

Receiver operating characteristic (ROC) curve analysis was performed to evaluate the ability of the CALLY index to discriminate CMV-positive patients from their CMV-negative counterparts. The area under the curve (AUC), optimal cut-off value according to the Youden index, sensitivity, specificity, positive predictive value, and negative predictive value were calculated.

Univariable logistic regression analyses were initially performed to identify variables associated with tissue-confirmed CMV colitis. Variables with a *p* value < 0.10 in univariable analysis, together with clinically relevant covariates identified from previous literature, were considered for inclusion in the multivariable logistic regression model. Disease extent was entered as extensive colitis (Montreal E3) versus non-E3 disease (E1–E2). Adjusted odds ratios (ORs) and 95% confidence intervals (CIs) are reported. Potential multicollinearity among variables included in the multivariable model was assessed using variance inflation factors (VIFs). All VIF values were below 2.0, indicating no evidence of significant multicollinearity.

Treatment-related variables were not included in the primary multivariable model because of the limited number of CMV-positive events and the potential for overfitting. In addition, treatment exposure was considered to be closely related to disease severity and treatment indication, which could introduce indication bias.

All statistical tests were two-sided, and a *p* value < 0.05 was considered statistically significant.

### 2.7. Ethical Approval

The study was conducted in accordance with the Declaration of Helsinki and approved by the Bursa Uludağ University Clinical Research Ethics Committee (Approval No. 2023-10/36; 10 October 2023).

## 3. Results

### 3.1. Baseline Characteristics

A total of 82 patients with active ulcerative colitis were included in the study. Tissue-confirmed CMV colitis was identified in 28 patients (34.1%), whereas 54 patients (65.9%) were classified as CMV-negative. The baseline demographic, clinical, and treatment characteristics of the study population are summarized in Table 1.

Among the 28 patients classified as having tissue-confirmed CMV colitis, characteristic CMV cytopathic inclusions on routine histopathological examination were identified in 2 patients (7.1%), whereas CMV immunohistochemical staining was positive in all 28 patients (100%). Tissue CMV-PCR was also positive in all CMV-positive patients and served as supportive virological evidence in accordance with the institutional diagnostic protocol.

CMV-positive patients were significantly older than CMV-negative patients [55.5 (40.5–63.8) vs. 37.5 (26.2–53.8) years, *p* = 0.009] and had a longer disease duration [60.0 (45.0–98.5) vs. 47.0 (39.2–58.8) months, *p* = 0.022]. Disease activity was significantly greater in the CMV-positive group, as reflected by higher total Mayo scores [10.0 (9.0–11.0) vs. 9.0 (8.0–10.0), *p* = 0.003], a higher frequency of severe clinical disease (92.9% vs. 57.4%, *p* = 0.002), and a greater prevalence of MES 3 lesions (82.1% vs. 50.0%, *p* = 0.017).

CMV-positive patients more frequently exhibited pancolitis/Montreal E3 disease (71.4% vs. 33.3%, *p* = 0.002). Anti-TNF exposure was substantially more common among CMV-positive patients (96.4% vs. 51.9%, *p* < 0.001), whereas vedolizumab or ustekinumab use was less frequent (3.6% vs. 31.5%, *p* = 0.009).

### 3.2. Laboratory Findings and CALLY Index

Compared with CMV-negative patients, those with CMV colitis had significantly lower serum albumin concentrations [29.5 (25.5–34.3) vs. 34.5 (30.3–38.0) g/L, *p* < 0.001], lower hemoglobin levels [10.6 (9.2–11.8) vs. 11.6 (10.6–12.3) g/dL, *p* = 0.039], and markedly elevated fecal calprotectin concentrations [440 (369–563) vs. 252 (201–326) µg/g, *p* < 0.001]. White blood cell counts were numerically higher in CMV-positive patients [8.55 (7.17–10.17) vs. 7.68 (6.32–8.85) ×10^9^/L], although the difference did not reach statistical significance (*p* = 0.065).

No significant differences were observed in CRP (*p* = 0.089), ESR (*p* = 0.961), platelet count (*p* = 0.216), or absolute lymphocyte count (*p* = 0.353). Although median CALLY index values were numerically higher in CMV-positive patients [5957.9 (3415.8–9124.6) vs. 2814.6 (1119.4–12,013.1)], this difference did not reach statistical significance (*p* = 0.136).

Comparisons of laboratory findings and CALLY index values between CMV-positive and CMV-negative patients are summarized in Table 2.

### 3.3. Diagnostic Performance of the CALLY Index

The diagnostic performance of the CALLY index and other selected biomarkers for identifying tissue-confirmed CMV colitis is summarized in Table 3.

**Figure 2:**
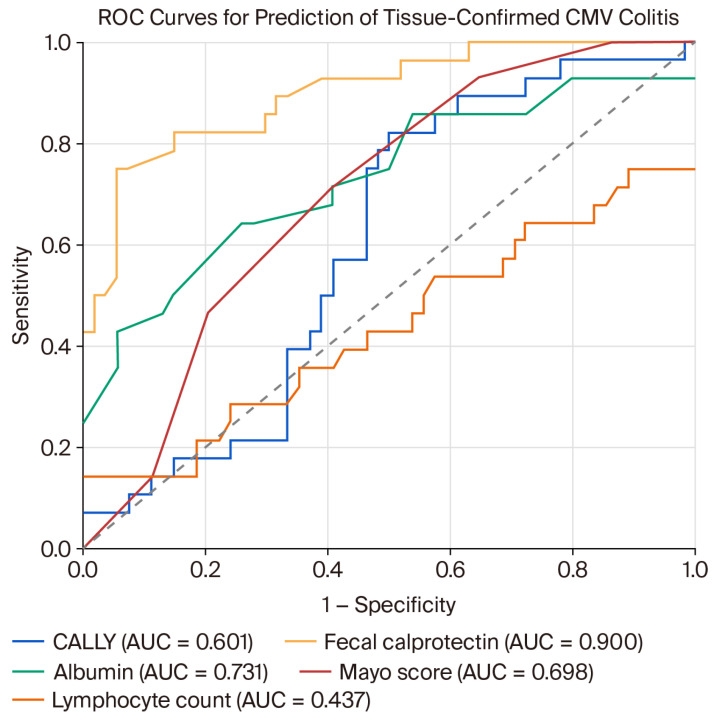
Receiver operating characteristic (ROC) curves of the CALLY index, fecal calprotectin, albumin, Mayo score, and lymphocyte count for identifying tissue-confirmed CMV colitis.

ROC curve analysis demonstrated limited discriminative performance of the CALLY index for identifying tissue-confirmed CMV colitis (Figure 2), with an AUC of 0.601 (95% CI: 0.472–0.722, *p* = 0.136). The optimal cut-off value determined by the Youden index was 2838, providing a sensitivity of 82.1% and a specificity of 50.0%. At this threshold, the CALLY index yielded a positive predictive value of 46.0% and a negative predictive value of 84.4%.

Among all evaluated biomarkers, fecal calprotectin demonstrated the highest diagnostic performance (AUC = 0.900, 95% CI: 0.819–0.962, *p* < 0.001), followed by albumin (AUC = 0.731, 95% CI: 0.597–0.851, *p* < 0.001) and total Mayo score (AUC = 0.698, 95% CI: 0.584–0.805, *p* = 0.003), whereas the CALLY index showed only modest discriminative ability (AUC = 0.601), consistent with its limited standalone diagnostic performance. Absolute lymphocyte count showed poor discriminatory performance (AUC = 0.437, *p* = 0.353).

Variables associated with tissue-confirmed CMV colitis in univariable and multivariable logistic regression analyses are summarized in Table 4.

### 3.4. Logistic Regression Analysis

In univariable logistic regression analysis, age, Mayo score, extensive colitis, albumin level, fecal calprotectin, and a CALLY index ≥ 2838 were significantly associated with CMV positivity.

In the multivariable model adjusted for age, disease activity, disease extent, albumin level, and fecal calprotectin, a CALLY index ≥ 2838 remained independently associated with tissue-confirmed CMV colitis (OR = 32.09, 95% CI: 3.27–315.39, *p* = 0.003). Fecal calprotectin also remained independently associated with tissue-confirmed CMV colitis (OR = 1.021, 95% CI: 1.009–1.034, *p* = 0.001), whereas age, Mayo score, extensive colitis, and albumin lost statistical significance after adjustment.

As a sensitivity analysis, the association between CALLY ≥ 2838 and tissue-confirmed CMV colitis remained statistically significant after additional adjustment for anti-TNF exposure (OR 4.15, 95% CI 1.25–13.73; *p* = 0.020).

### 3.5. Summary of Findings

Although the CALLY index demonstrated limited standalone diagnostic performance, a CALLY value ≥ 2838 was independently associated with tissue-confirmed CMV colitis after adjustment for major clinical and inflammatory confounders. These findings suggest that a CALLY value ≥ 2838 was associated with tissue-confirmed CMV colitis in this cohort. However, because of the modest discriminative performance, the wide confidence interval of the adjusted odds ratio, and the lack of external validation, this finding should be considered exploratory and requires confirmation in larger independent cohorts before clinical application.

## 4. Discussion

In the present study, we evaluated the association between the C-reactive protein–albumin–lymphocyte (CALLY) index and tissue-confirmed CMV colitis in patients with active ulcerative colitis. Although the CALLY index demonstrated limited discriminative performance as a standalone diagnostic marker, a CALLY value ≥ 2838 remained independently associated with tissue-confirmed CMV colitis after adjustment for established clinical and laboratory risk factors. In addition, CMV-positive patients exhibited significantly greater disease activity, more extensive colonic involvement, lower albumin levels, and markedly elevated fecal calprotectin concentrations. These findings are consistent with previous studies demonstrating that severe disease activity, extensive colitis, and markers of systemic inflammation are closely associated with CMV reactivation in ulcerative colitis. A recent meta-analysis by Qin et al. identified severe UC, pancolitis, corticosteroid exposure, and immunosuppressive therapy as major risk factors for CMV reactivation, supporting the concept that CMV infection preferentially occurs in patients with a high inflammatory burden [5]. Similarly, Altunal et al. reported that inflammatory and nutritional parameters may provide early clues to CMV reactivation [18], while Lin et al. demonstrated the prognostic value of inflammation-based indices in assessing disease severity and progression in ulcerative colitis [15]. Similarly, Zhang et al. demonstrated that tissue-confirmed CMV colitis was associated with significantly poorer short- and long-term clinical outcomes in patients with ulcerative colitis, highlighting the clinical importance of early recognition and appropriate diagnostic evaluation of CMV infection [22]. Collectively, these observations provide a potential biological explanation for the observed association between the CALLY index and CMV colitis in our cohort.

One of the most notable findings of the present study was the association between higher CALLY values and CMV positivity. Although the CALLY index itself did not demonstrate strong standalone diagnostic performance, its relationship with CMV colitis may be explained by the biological significance of its individual components. In our cohort, CMV-positive patients exhibited significantly lower serum albumin concentrations compared with CMV-negative patients, whereas absolute lymphocyte counts did not differ significantly between groups. Previous studies have consistently identified hypoalbuminemia as an important marker of CMV infection and reactivation in ulcerative colitis. Yang et al. demonstrated that reduced albumin levels were among the earliest indicators of CMV infection in patients with acute ulcerative colitis [19], while the meta-analysis by Qin et al. confirmed hypoalbuminemia as a significant risk factor for CMV reactivation [5]. Low albumin levels likely reflect a combination of severe systemic inflammation, impaired nutritional status, and increased intestinal protein loss, all of which are commonly observed in patients with severe UC. Consequently, the association between the CALLY index and CMV positivity may partly reflect the contribution of albumin as an integrated marker of disease burden and inflammatory activity. However, the persistence of CALLY as an independent predictor after adjustment for albumin in multivariable analysis suggests that the composite nature of the index may provide additional information beyond albumin alone. Because serum albumin is a component of the CALLY index, the possibility of multicollinearity was specifically evaluated during model construction. Variance inflation factor analysis demonstrated no evidence of significant multicollinearity, supporting the inclusion of both variables in the multivariable model.

The potential utility of the CALLY index in CMV colitis may also be related to its ability to integrate multiple pathophysiological domains into a single composite marker. Unlike conventional inflammatory biomarkers, the CALLY index simultaneously reflects systemic inflammation, nutritional status, and immune competence through the combined assessment of C-reactive protein, albumin, and lymphocyte count. Nevertheless, our findings also demonstrated that fecal calprotectin exhibited substantially greater diagnostic performance than the CALLY index for identifying CMV-positive patients. This observation is consistent with previous studies showing that fecal calprotectin closely reflects intestinal inflammatory activity and correlates strongly with endoscopic disease severity in ulcerative colitis [23]. Recent studies have demonstrated the prognostic significance of the CALLY index across diverse clinical settings, including both malignant and chronic inflammatory diseases. For example, Zhang et al. reported a significant association between the CALLY index and all-cause mortality in patients with rheumatoid arthritis, suggesting that the index may capture fundamental biological processes related to chronic inflammation and immune dysregulation [24]. Taken together, these findings suggest that while the CALLY index may provide complementary information regarding systemic inflammatory burden, it should not be considered a substitute for established markers of intestinal inflammation such as fecal calprotectin.

Despite the independent association observed in multivariable analysis, the CALLY index demonstrated limited discriminative performance when evaluated as a standalone diagnostic marker. This finding may reflect the complex pathophysiology of CMV reactivation in ulcerative colitis. CMV infection is not solely driven by systemic inflammation but rather results from a multifaceted interaction between viral latency, host immune regulation, inflammatory activity, and exposure to immunosuppressive therapies [20]. Previous studies have emphasized that CMV may act either as a true pathogen or as a bystander reflecting severe underlying inflammation, further complicating the identification of patients with clinically significant CMV disease [6,7]. Consequently, no single laboratory parameter is likely to fully capture the biological complexity of CMV reactivation. The modest diagnostic performance observed in our study therefore suggests that the CALLY index should be considered a complementary risk stratification tool rather than a definitive diagnostic marker for CMV colitis.

Another noteworthy finding was the discrepancy between diagnostic discrimination and risk association. ROC analysis demonstrated only modest discriminatory performance of the CALLY index, with an AUC of 0.601. In contrast, multivariable logistic regression identified CALLY ≥ 2838 as independently associated with tissue-confirmed CMV colitis after adjustment for major clinical confounders. Although these findings may initially appear contradictory, diagnostic discrimination and risk association represent distinct statistical concepts. ROC analysis evaluates the ability of a biomarker to accurately classify individual patients, whereas logistic regression quantifies the strength of association between a variable and an outcome after accounting for other covariates. Previous methodological studies have demonstrated that biomarkers associated with large odds ratios may nevertheless exhibit limited discriminatory performance when applied at the individual patient level [25]. Therefore, our findings suggest that although the CALLY index is not sufficiently accurate to function as a standalone diagnostic test, it may still capture clinically relevant biological information associated with CMV reactivation despite its limited ability to discriminate between individual patients.

Although the multivariable analysis demonstrated an independent association between the dichotomized CALLY index and tissue-confirmed CMV colitis, the wide confidence interval indicates limited precision of the estimated effect size, most likely reflecting the relatively small number of CMV-positive patients. Therefore, the magnitude of the association should be interpreted cautiously.

Our ROC analysis also demonstrated that fecal calprotectin was the strongest individual predictor of CMV colitis, achieving an AUC of 0.900. This finding is biologically plausible given the close relationship between CMV infection and severe mucosal inflammation. Albumin and Mayo score also showed better discriminative performance than the CALLY index. Previous studies have demonstrated that fecal calprotectin closely reflects endoscopic disease activity and mucosal inflammatory burden in ulcerative colitis [23]. These observations indicate that the principal clinical utility of the CALLY index may not lie in replacing established inflammatory biomarkers but rather in providing complementary information regarding the interaction between inflammation, nutrition, and immune status.

The present study has several limitations. First, its retrospective single-center design may have introduced selection bias and limits the generalizability of our findings. Second, although the study included a clinically well-defined cohort with tissue-confirmed CMV assessment, the relatively small sample size, particularly the limited number of CMV-positive patients, may have reduced statistical precision and contributed to the wide confidence intervals observed in the multivariable analyses. In addition, treatment-related variables such as anti-TNF exposure were not incorporated into the primary multivariable model because of the limited number of CMV-positive events and the risk of model overfitting. Furthermore, treatment exposure is closely related to disease severity and therapeutic indication. Therefore, residual confounding by treatment differences cannot be excluded and may have contributed, at least in part, to the observed association between the CALLY index and tissue-confirmed CMV colitis. Therefore, the magnitude of the estimated effect sizes, particularly for the CALLY index, should be interpreted with appropriate caution. Third, because of the cross-sectional design, causal relationships between the CALLY index and CMV colitis cannot be established. Finally, the proposed CALLY cut-off was derived from a single-center cohort and has not undergone external validation. Consequently, its clinical applicability should be considered exploratory until confirmed in larger, prospective, multicenter studies.

Although treatment-related variables were not included in the primary multivariable model because of the limited number of CMV-positive events and the risk of model overfitting, a sensitivity analysis additionally adjusting for anti-TNF exposure yielded similar findings, with CALLY ≥ 2838 remaining independently associated with tissue-confirmed CMV colitis (OR 4.15, 95% CI 1.25–13.73; *p* = 0.020). Nevertheless, residual confounding related to treatment indication and disease severity cannot be completely excluded.

Despite these limitations, the present study possesses several important strengths. CMV status was determined using tissue-confirmed diagnostic methods, including histopathology, immunohistochemistry, and tissue CMV DNA analysis, rather than blood-based assays alone, thereby reducing the risk of outcome misclassification. In addition, comprehensive clinical, laboratory, endoscopic, and treatment-related data were available, allowing adjustment for multiple potential confounders. To our knowledge, this is the first study specifically evaluating the relationship between the CALLY index and tissue-confirmed CMV colitis in patients with active ulcerative colitis.

## 5. Conclusions

In conclusion, the C-reactive protein–albumin–lymphocyte (CALLY) index was independently associated with tissue-confirmed CMV colitis in this clinically selected, high-risk cohort of patients with active ulcerative colitis. However, the CALLY index demonstrated only modest discriminative performance as a standalone diagnostic marker. Therefore, these findings should be considered exploratory and require validation in larger prospective multicenter studies before the CALLY index can be considered for routine clinical application.

## Figures and Tables

**Table 1:** Baseline demographic, clinical, and treatment characteristics according to tissue-confirmed CMV status.

Variable	CMV-Negative (*n* = 54)	CMV-Positive (*n* = 28)	*p* Value
Age, years	37.5 (26.2–53.8)	55.5 (40.5–63.8)	0.009
Male sex, *n* (%)	36 (66.7)	19 (67.9)	1.000
Disease duration, months	47.0 (39.2–58.8)	60.0 (45.0–98.5)	0.022
Total Mayo score	9.0 (8.0–10.0)	10.0 (9.0–11.0)	0.003
**Clinical disease severity, *n* (%)**			**0.002**
Moderate	23 (42.6)	2 (7.1)	
Severe	31 (57.4)	26 (92.9)	
**Mayo Endoscopic Subscore, *n* (%)**			**0.017**
MES 1	1 (1.9)	0 (0.0)	
MES 2	26 (48.1)	5 (17.9)	
MES 3	27 (50.0)	23 (82.1)	
**Disease extent, *n* (%)**			**0.001**
Left-sided colitis	10 (18.5)	5 (17.9)	
Extensive colitis	26 (48.1)	3 (10.7)	
Pancolitis	18 (33.3)	20 (71.4)	
Pancolitis/Montreal E3, *n* (%)	18 (33.3)	20 (71.4)	0.002
Corticosteroid exposure, *n* (%)	54 (100.0)	28 (100.0)	NA
Azathioprine use, *n* (%)	45 (83.3)	24 (85.7)	1.000
Anti-TNF therapy, *n* (%)	28 (51.9)	27 (96.4)	<0.001
Vedolizumab or ustekinumab use, *n* (%)	17 (31.5)	1 (3.6)	0.009

Data are presented as median (interquartile range) or *n* (%). CMV: cytomegalovirus; MES: Mayo Endoscopic Subscore; TNF: tumor necrosis factor; NA: not applicable. *p* values were calculated using the Mann–Whitney U test for continuous variables and chi-square or Fisher’s exact tests for categorical variables, as appropriate.

**Table 2:** Laboratory parameters and components of the CALLY index according to CMV status.

Variable	CMV-Negative (*n* = 54)	CMV-Positive (*n* = 28)	*p* Value
C-reactive protein (CRP), mg/L	23.5 (7.3–56.8)	13.5 (6.0–21.5)	0.089
Erythrocyte sedimentation rate (ESR), mm/h	32.5 (18.0–45.8)	30.0 (19.3–49.0)	0.961
Albumin, g/L	34.5 (30.3–38.0)	29.5 (25.5–34.3)	<0.001
Hemoglobin, g/dL	11.6 (10.6–12.3)	10.6 (9.2–11.8)	0.039
White blood cell count, ×10^9^/L	7.79 (6.32–8.85)	8.55 (7.17–10.17)	0.065
Absolute lymphocyte count, µL	2150 (1493–2968)	2320 (1431–3745)	0.353
Platelet count, ×10^9^/L	342 (287–412)	394 (248–550)	0.216
Fecal calprotectin, µg/g	252 (201–326)	440 (369–563)	<0.001
CALLY index	2814.6 (1119.4–12,013.1)	5957.9 (3415.8–9124.6)	0.136

Data are presented as median (interquartile range). Abbreviations: CMV, cytomegalovirus; CRP, C-reactive protein; ESR, erythrocyte sedimentation rate; CALLY, C-reactive protein–albumin–lymphocyte index.

**Table 3:** Diagnostic performance of the CALLY index and selected biomarkers for identifying tissue-confirmed CMV colitis. Comparators were selected because they represent established clinical, inflammatory, and disease activity markers that have been associated with cytomegalovirus infection or disease severity in patients with active ulcerative colitis.

Predictor	AUC (95% CI)	Optimal Cut-Off	Direction	Sensitivity (%)	Specificity (%)	PPV (%)	NPV (%)	Youden Index	*p* Value
CALLY index	0.601 (0.472–0.722)	2838	≥	82.1	50.0	46.0	84.4	0.321	0.136
C-reactive protein (CRP), mg/L	0.615 (0.490–0.727)	48.0	≤	96.4	33.3	42.9	94.7	0.298	0.089
Albumin, g/L	0.731 (0.597–0.851)	30.0	≤	64.3	74.1	56.3	80.0	0.384	<0.001
Absolute lymphocyte count, /µL	0.437 (0.414–0.706)	4180	≥	25.0	100.0	100.0	72.0	0.250	0.353
Fecal calprotectin, µg/g	0.900 (0.819–0.962)	375	≥	75.0	94.4	87.5	87.9	0.694	<0.001
Total Mayo score	0.698 (0.584–0.805)	10	≥	71.4	59.3	47.6	80.0	0.307	0.003

Data are derived from ROC curve analysis for the discrimination of tissue-confirmed CMV-positive and CMV-negative patients. Optimal cut-off values were determined using the Youden index. For variables in which lower values indicated tissue-confirmed CMV colitis, the direction of the threshold is shown as ≤. For variables in which higher values indicated higher probability of CMV positivity, the direction is shown as ≥. Abbreviations: AUC, area under the receiver operating characteristic curve; CI, confidence interval; CALLY, C-reactive protein–albumin–lymphocyte index; CMV, cytomegalovirus; CRP, C-reactive protein, PPV, positive predictive value; NPV, negative predictive value.

**Table 4:** Univariable and multivariable logistic regression analysis of factors associated with tissue-confirmed CMV colitis.

Variable	Univariable OR (95% CI)	*p* Value	Multivariable OR (95% CI)	*p* Value
Age (per year)	1.04 (1.01–1.07)	0.011	1.02 (0.97–1.07)	0.467
Mayo score	1.65 (1.16–2.34)	0.005	1.35 (0.70–2.60)	0.373
Disease extent (Montreal E3 vs. non-E3)	2.12 (1.07–4.19)	0.031	1.24 (0.40–3.83)	0.711
Albumin (per g/L)	0.86 (0.79–0.95)	0.002	0.91 (0.78–1.06)	0.213
Fecal calprotectin (per µg/g)	1.017 (1.009–1.025)	<0.001	1.021 (1.009–1.034)	0.001
CALLY ≥ 2838	4.60 (1.52–13.88)	0.007	32.09 (3.27–315.39)	0.003

Abbreviations: OR, odds ratio; CI, confidence interval; CALLY, C-reactive protein–albumin–lymphocyte index. Multivariable model adjusted for age, Mayo score, disease extent, albumin level, and fecal calprotectin. Statistically significant associations are defined as *p* < 0.05.

## Data Availability

Due to privacy and ethical restrictions involving human participant data, the datasets generated and analyzed during the current study are not publicly available. De-identified data may be made available by the corresponding author upon reasonable request and subject to approval by the institutional ethics committee.

## References

[1] Sheriff M.Z., Mansoor E., Luther J., Ananthakrishnan A.N., Abou Saleh M., Ho E., Briggs F.B.S., Dave M. (2020). Opportunistic infections are more common in Crohn’s disease and ulcerative colitis: A large population-based study. Inflamm. Bowel Dis..

[2] Soni K., Puing A. (2025). Cytomegalovirus colitis in adults with inflammatory bowel disease. Viruses.

[3] Shukla T., Singh S., Loftus E.V., Bruining D.H., McCurdy J.D. (2015). Antiviral therapy in steroid-refractory ulcerative colitis with cytomegalovirus: Systematic review and meta-analysis. Inflamm. Bowel Dis..

[4] Lee H.S., Park S.H., Kim S.H., Kim J., Choi J., Lee H.J., Kim W.S., Lee J.-M., Kwak M.S., Hwang S.W. (2016). Risk factors and clinical outcomes associated with cytomegalovirus colitis in patients with acute severe ulcerative colitis. Inflamm. Bowel Dis..

[5] Qin Y., Wang G., Kong D., Li G., Wang H., Qin H., Wang H. (2021). Risk factors of cytomegalovirus reactivation in ulcerative colitis patients: A meta-analysis. Diagnostics.

[6] Jentzer A., Veyrard P., Roblin X., Saint-Sardos P., Rochereau N., Paul S., Bourlet T., Pozzetto B., Pillet S. (2020). Cytomegalovirus and inflammatory bowel diseases (IBD) with a special focus on the Link with ulcerative colitis (UC). Microorganisms.

[7] Lawlor G., Moss A.C. (2010). Cytomegalovirus in inflammatory bowel disease: Pathogen or innocent bystander?. Inflamm. Bowel Dis..

[8] Sager K., Alam S., Bond A., Chinnappan L., Probert C.S. (2015). Review article: Cytomegalovirus and inflammatory bowel disease. Aliment. Pharmacol. Ther..

[9] Park S.C., Jeen Y.M., Jeen Y.T. (2017). Approach to cytomegalovirus infections in patients with ulcerative colitis. Korean J. Intern. Med..

[10] Esen S., Saglik I., Dolar E., Cesur S., Ugras N., Agca H., Merdan O., Ener B. (2024). Diagnostic utility of cytomegalovirus (CMV) DNA quantitation in ulcerative colitis. Viruses.

[11] Nowacki T.M., Bettenworth D., Meister T., Heidemann J., Lenz F., Schmidt H.H., Heinzow H.S. (2018). Novel score predicts risk for cytomegalovirus infection in ulcerative colitis. J. Clin. Virol..

[12] Li J., Zhang S., Hu X., Huang T., Chen M. (2025). Correlation between the C-reactive protein (CRP)-albumin-lymphocyte (CALLY) index and the prognosis of gastric cancer patients after gastrectomy: A systematic review and meta-analysis. Surg. Today.

[13] Xu Z., Tang J., Chen X., Jin Y., Zhang H., Liang R. (2024). Associations of the C-reactive protein-albumin-lymphocyte (CALLY) index with cardiorenal syndrome: Insights from a population-based study. Heliyon.

[14] Yang M., Lin S.Q., Liu X.Y., Tang M., Hu C.-L., Wang Z.-W., Zhang Q., Zhang X., Song M.-M., Ruan G.-T. (2023). Association between C-reactive protein-albumin-lymphocyte (CALLY) index and overall survival in patients with colorectal cancer: From the investigation on nutrition status and clinical outcome of common cancers study. Front. Immunol..

[15] Lin H., Bai Z., Wu Q., Chu G., Zhang Y., Guo X., Qi X. (2022). Inflammatory indices for assessing ulcerative colitis severity and disease progression: A single-center retrospective study. Front. Public Health.

[16] Con D., Andrew B., Nicolaides S., van Langenberg D.R., Vasudevan A. (2022). Biomarker dynamics during infliximab rescue therapy in acute severe ulcerative colitis: CRP-lymphocyte ratio and CRP-albumin ratio are useful in predicting colectomy. Intest. Res..

[17] Grant R.K., Jones G.R., Plevris N., Lynch R.W., Jenkinson P.W., Lees C.W., Manship T.A., Jagger F.A.M., Brindle W.M., Shivakumar M. (2021). The ACE (Albumin, CRP and Endoscopy) index in acute colitis: A simple clinical index on admission that predicts outcome in patients with acute ulcerative colitis. Inflamm. Bowel Dis..

[18] Altunal L.N., Ozel A.S., Ak C. (2023). Cytomegalovirus reactivation: Early indicators in patients with ulcerative colitis. Niger. J. Clin. Pract..

[19] Yang H., Wu K., Zhang H., Owyang Q., Miao Y., Gu F., Hu N., Zou K., Sheng J., Li J. (2020). IgA, albumin and eosinopenia as early indicators of cytomegalovirus infection in patients with acute ulcerative colitis. BMC Gastroenterol..

[20] Forte E., Zhang Z., Thorp E.B., Hummel M. (2020). Cytomegalovirus latency and reactivation: An intricate interplay with the host immune response. Front. Cell. Infect. Microbiol..

[21] Mestrovic A., Perkovic N., Bozic D., Kumric M., Vilovic M., Bozic J. (2024). Precision medicine in inflammatory bowel disease: A spotlight on emerging molecular biomarkers. Biomedicines.

[22] Zhang M., Bai X., Zhang H., You Y., Lv H., Li Y., Tan B., Li J., Xu H., Zheng W. (2022). The role of cytomegalovirus colitis on short- and long-term outcomes for patients with ulcerative colitis. Scand. J. Gastroenterol..

[23] Kawashima K., Ishihara S., Yuki T., Fukuba N., Oshima N., Kazumori H., Sonoyama H., Yamashita N., Tada Y., Kusunoki R. (2016). Fecal calprotectin level correlated with both endoscopic severity and disease extent in ulcerative colitis. BMC Gastroenterol..

[24] Zhang J., Lin Y., Zeng J., Luo G., Liao P., Chen Q., Zhong H., Liang S., Zhou C., Yang B. (2025). The C-reactive protein (CRP)-albumin-lymphocyte (CALLY) index exhibits an L-shaped association with all-cause mortality in rheumatoid arthritis patients: A retrospective cohort study. BMC Rheumatol..

[25] Jakobsdottir J., Gorin M.B., Conley Y.P., Ferrell R.E., Weeks D.E. (2009). Interpretation of genetic association studies: Markers with replicated highly significant odds ratios may be poor classifiers. PLoS Genet..

